# Pineal Gland Tumour With Drop Metastases: A Case Report

**DOI:** 10.7759/cureus.29855

**Published:** 2022-10-03

**Authors:** Shelva Meena Gurusamy, Azhany Yaakub, Wan-Hazabbah Wan Hitam, Siti Aishah Ahmad Maulana, Wan Zulkafli Wan Ibrahim

**Affiliations:** 1 Department of Ophthalmology and Visual Science, Universiti Sains Malaysia School of Medical Sciences, Kelantan, MYS; 2 Department of Radiology, Faculty of Medicine and Health Sciences, Universiti Sultan Zainal Abidin, Terengganu, MYS; 3 Department of Radiology, Hospital Sultanah Nur Zahirah, Terengganu, MYS

**Keywords:** neurological manifestations, young, parinaud syndrome, germinoma, pineal gland tumor

## Abstract

Pineal gland tumours are reported rarely in Malayasia and early diagnosis and intervention promise a better prognosis for patients. We report a rare case of pineal gland tumour with drop metastases in the fourth ventricle in a 20-year-old young male with Parinaud syndrome. The patient, who had no underlying medical illnesses, presented with neurological symptoms and limb weakness associated with tremors and blurring of vision which worsened over a span of four months. The patient was having difficulty in ambulating with reduced power over the lower limbs with tremors as well as Parinaud syndrome indicated through the limitation of upward gaze, light-near dissociation of the pupils and convergence nystagmus. An MRI showed the presence of a pineal gland tumour with drop metastases in the fourth ventricle with calcification. The patient underwent an endoscopic third ventriculostomy and tumour biopsy. The biopsy indicated a pineal gland tumour with a germinoma subset and the patient was subjected to radiotherapy. Latency of diagnosis is an important prognostic factor as it reduces the survival rate for these patients hence the following discussion on the pineal gland tumour and its diagnostic dilemma.

## Introduction

The pineal gland is located in the midbrain in close proximity to the superior colliculus, situated in between both the cerebral hemisphere [1]. It is near the nucleus of Cajal and the rostral interstitial nucleus of the medial longitudinal fasciculus (riMLF). The nerve fibres go through to the vertical gaze nuclei, i.e. oculomotor and trochlear nuclei. It was previously thought to be a vestigial organ and has now been identified as a neuroendocrine organ responsible for the production of serotonin, melatonin and N, N-dimethyltryptamine which plays a major role in the regulation of the circadian rhythm [2].

The presentation of pineal gland tumours can vary; their development can be as early as a few months to a few years and the symptoms presented depends largely on the location and the extension of the tumour. Clinical symptoms can also differ according to the age of presentation. Pineal gland lesions in children can also present as abnormal pubertal development specifically in accelerated stages of puberty in both males and females. Due to lesions in the hypothalamic-pituitary axis, these patients can also present with vasopressin deficiency. Pineal gland-specific lesions are not pathognomonic of the gland. As the symptoms are not specific, patients do not present with sleeping problems; however, there have been reported cases where sleep dysfunction such as narcolepsy occurs [3]. Common intracranial tumour presentation such as acute headache is not as common in pineal gland tumour cases unless bleeding of the tumour is seen. Due to the extension and compression, the patient can also present with brain stem dysfunction. The close proximity to the vertical gaze centre in the midbrain and the horizontal gaze centre in the pons causes patients to present with Parinaud syndrome characterized by superior gaze palsy, light-near dissociation of pupil and convergence retraction nystagmus on attempted upward gaze [4,5].

The aim of this case report is to discuss the pineal gland tumour which is reported rarely in Malaysia as well as its presentation and the associated diagnostic dilemmas for treatment and prognostic factors.

## Case presentation

A 20-year-old Malay gentleman, with no underlying medical illness presented with a history of right upper limb unintentional tremors and motor weakness of four months duration prior to the first presentation. This was associated with gait imbalance and movement assistance. He also complained of diplopia more on the upward gaze which was intermittent and the patient could not quantify whether there was a presence of vertical or horizontal diplopia. There was also associated blurring of vision, generalized with no scotoma. There were no associated trauma and no eye pain or redness noted by the patient. He also denied the presence of any floaters, flashes, halos or any gaze provoked headaches.

He was first seen in a peripheral hospital, where computer tomography (CT) of the brain and magnetic resonance imaging (MRI) of the brain and spine was done showing the presence of a pineal gland tumour with no metastases to the spine. He was then referred to Hospital Universiti Sains Malaysia (HUSM) Neurosurgical Ophthalmology team for surgical intervention.

On examination, the visual acuity of the right eye (RE) was 6/6 and the left eye (LE) was 6/6. Regarding optic nerve function, light brightness was 100% over the bilateral eye; red desaturation was not affected in both eyes and there was no relative afferent pupillary defect (RAPD). Colour vision was done using the Standard Ishihara chart, which was highest for both eyes scoring 15/15 showing no compromise of the optic nerve function.

There was obvious right eye hypotropia and limited extraocular muscle movement of bilateral eyes. Both eyes had a limitation of movement of the upward gaze of -2. Other movements of abduction, adduction and downward gazes of both eyes were not affected. Diplopia was noted in all gazes except downward gaze. There was the presence of positive convergence retraction. The pupillary reflex in light was poor but constricted well during accommodation. There was a presence of light-near dissociation. Otherwise, the anterior segment examination was normal with intraocular pressure of 12 mmHg for both eyes. The bilateral eyes’ fundus revealed a pink optic disc with a clear disc margin and good vessel calibre. The foveal reflex was good with a flat macula and normal surrounding retina. Systemic examination was normal, with all cranial nerves intact. Neurological examination showed normal tone for all upper and lower limbs with a full power of 5/5; however, there was hyperreflexia in all major joints for both upper and lower limbs. The sensation was intact for all limbs. There were no associated cerebellar signs.

**Figure 1:**
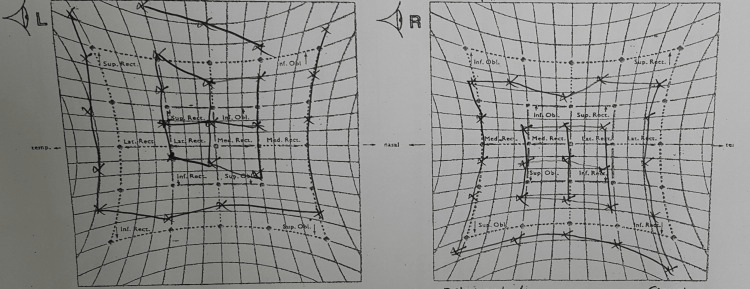
Hess test showing right eye hypotropia with limitation of upward gaze. There is compensatory upward gaze in the left eye

The baseline visual field examination was done and there was no visual field defect seen. Hess test for objective assessment of the extraocular muscle mobility was done, as seen in Figure 1 showing right eye hypotropia with upward gaze limitation.

**Figure 2:**
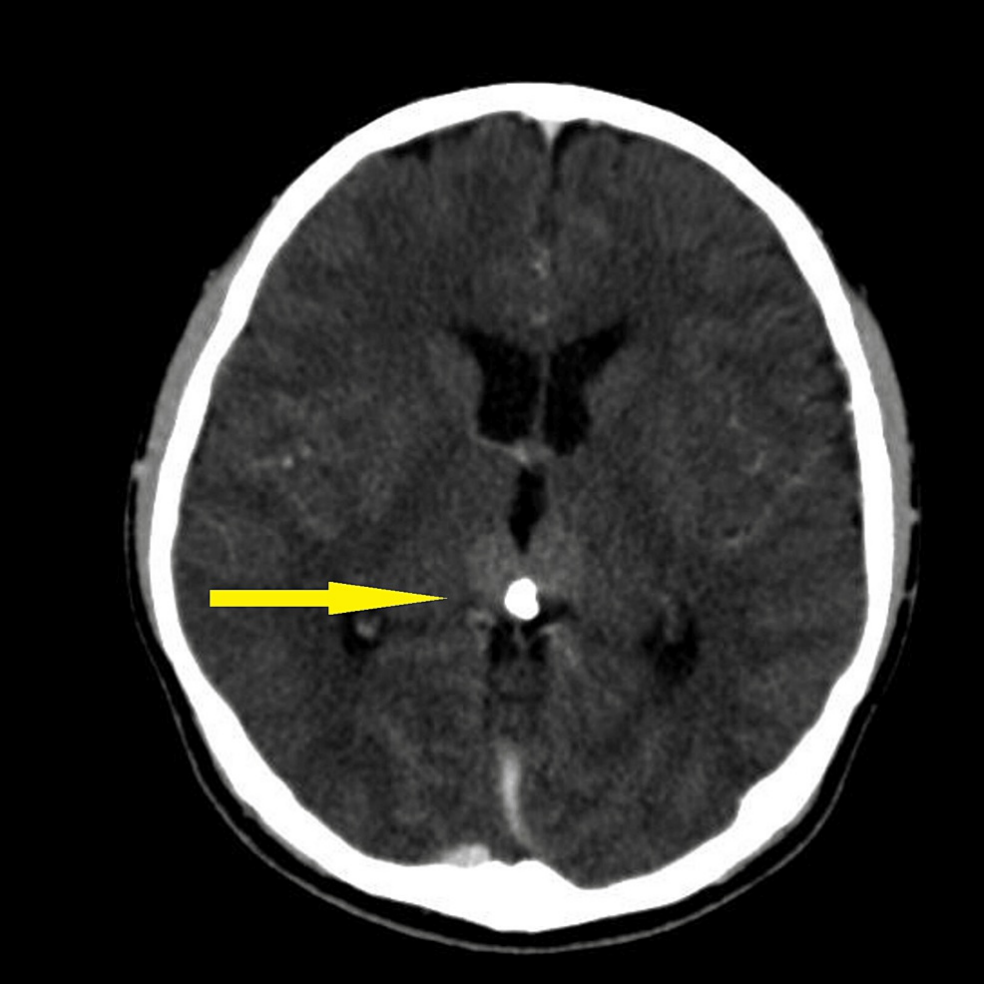
CT brain axial showing hyperdense lesion in the pineal gland region measuring 2.2 cm x 2.6 cm x 1.8 cm (yellow arrow).

**Figure 3:**
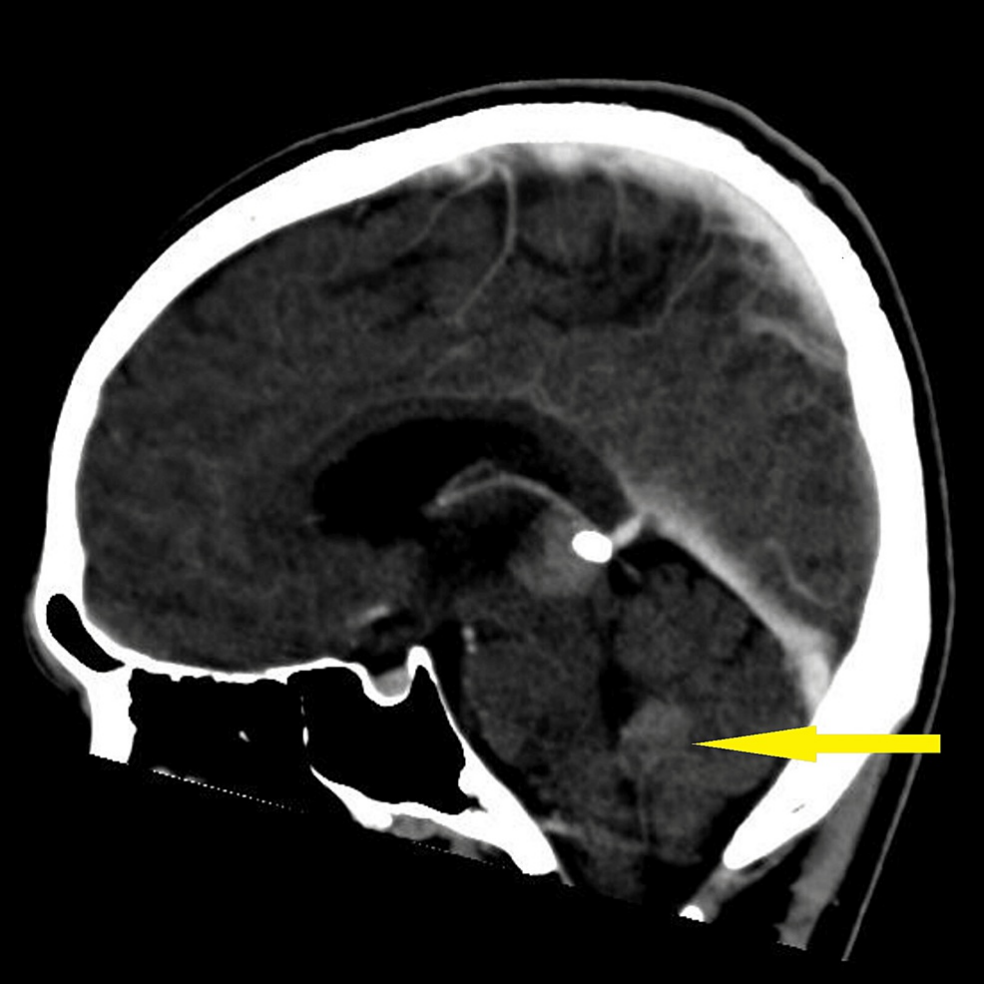
CT brain sagittal showing hypodense lesion in the right lateral and posterior wall of the fourth ventricle measuring 2.1 cm x 3.3 cm x 2.2 cm (yellow arrow)

**Figure 4:**
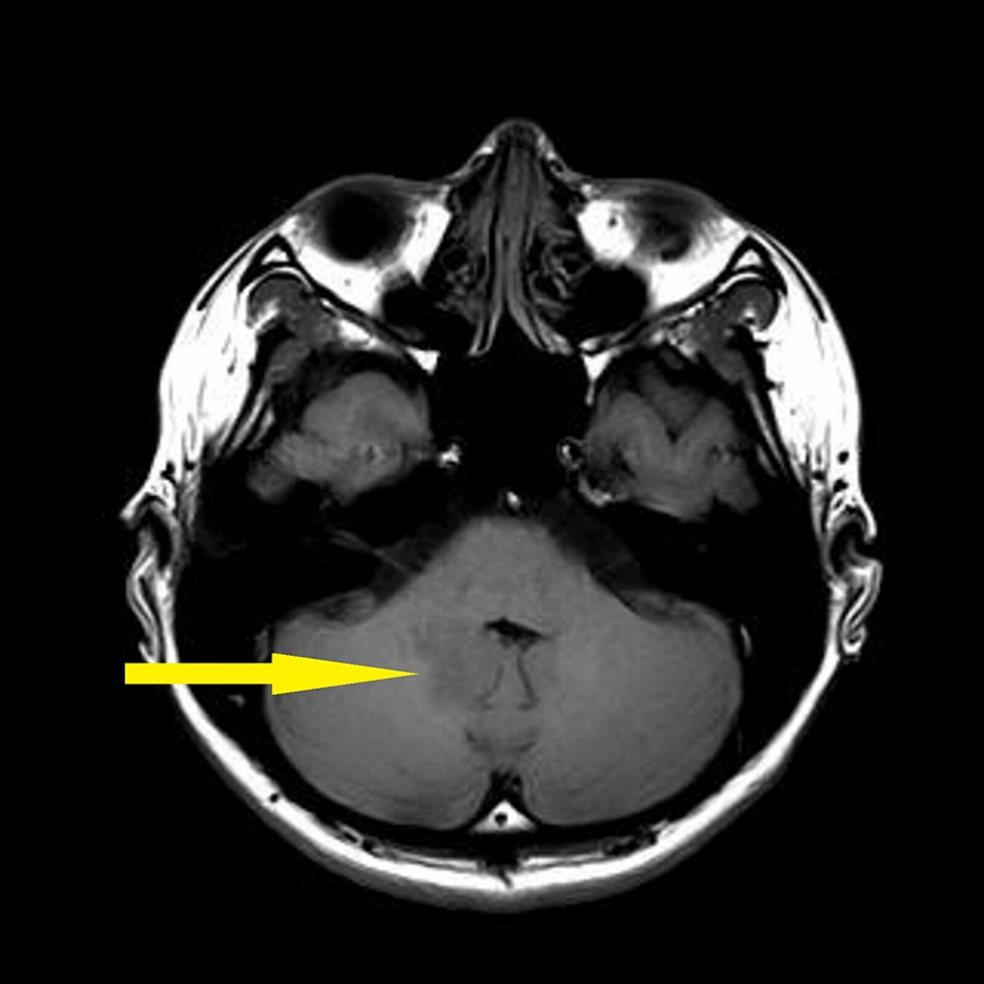
MRI brain (T1) axial showing an ill-defined lesion in the fourth ventricle, appearing isointense (yellow arrow)

**Figure 5:**
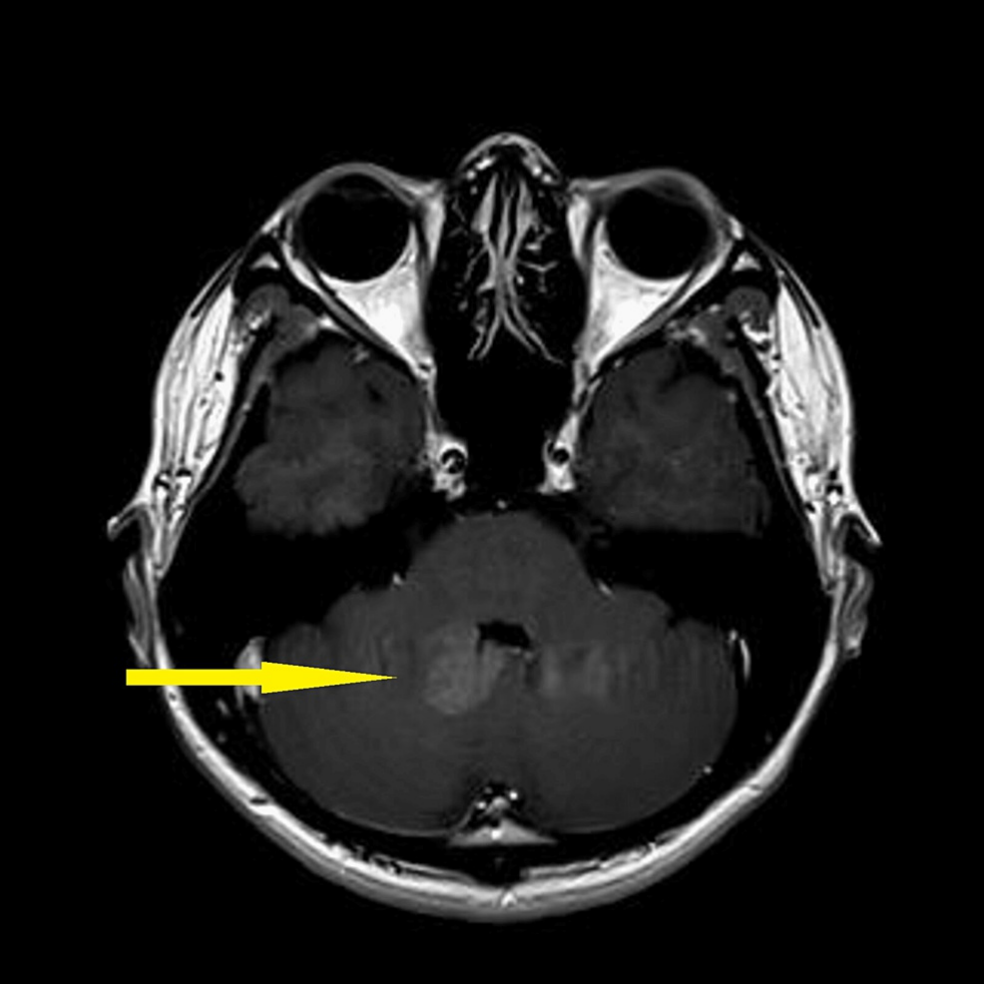
Enhanced MRI brain (T1) axial post-contrast showing an ill-defined lesion in the fourth ventricle (yellow arrow)

**Figure 6:**
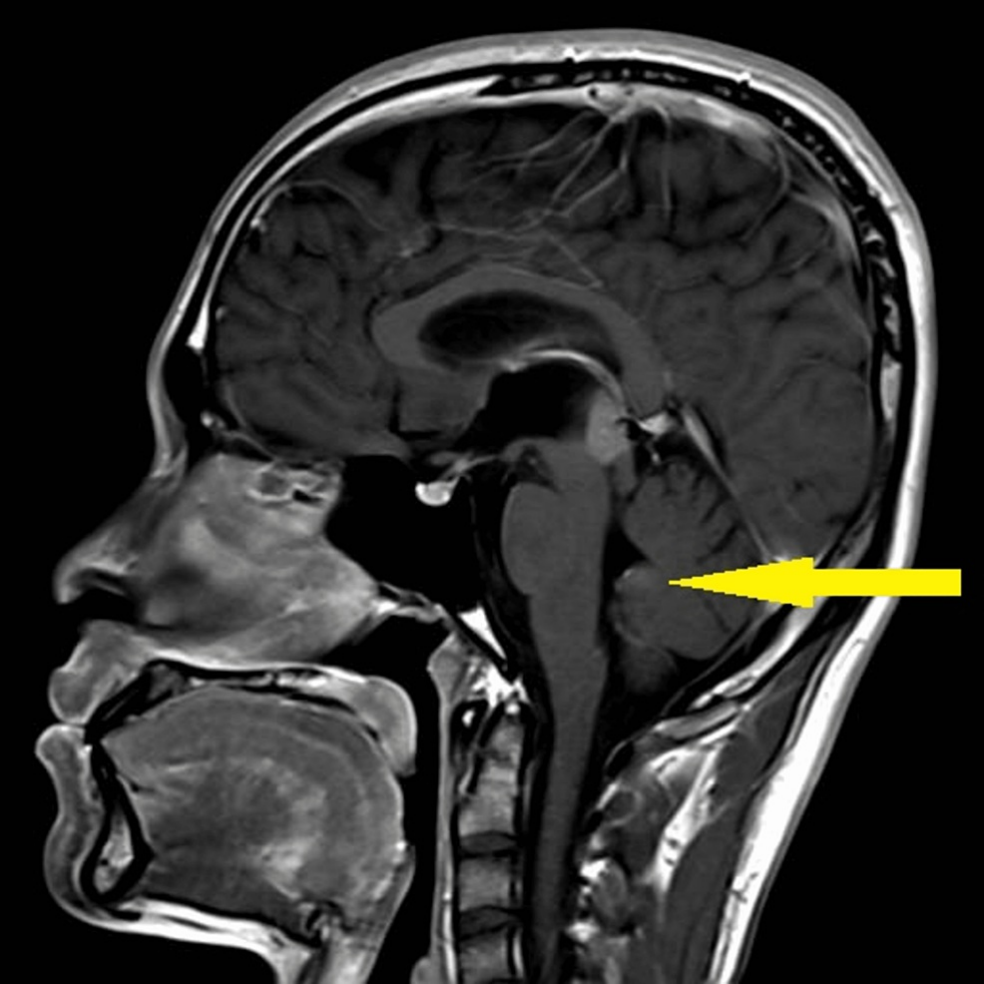
MRI brain (T1) sagittal showing an ill-defined lesion measuring 2.1 cm x 2.1 cm x 1.7 cm (yellow arrow)

Baseline blood investigation of tumour markers such as cancer antigen (CA) 125, alpha-fetoprotein (AFP), and beta-human chorionic gonadotropin (b-HCG) was found to be normal. The carcinoembryonic antigen (CEA) was slightly elevated to 3.5 compared to the normal value of less than 3. CT brain and MRI brain and spine (Figures 2-6) were done in the primary centre and the presence of a hyperdense mass in the pineal gland with densely calcified nodule was seen at its posterior aspect. A similar lesion was seen involving the right lateral and posterior wall of the fourth ventricle. There was no hydrocephalus seen. The spine was normal and there was no infiltration or masses.

The patient underwent an endoscopic third ventriculostomy and tumour biopsy. The tissue biopsy showed a pineal gland tumour of the germinoma subset. Tumour cells were positive for tumour marker CD117. The patient was then subsequently referred to the oncology team for radiotherapy. The patient responded well to chemotherapy and radiotherapy and is now currently on a six-monthly follow-up schedule to monitor his general status and ocular condition.

## Discussion

Pineal gland tumours are rare tumours comprising about 0.5% of all central nervous system tumours in adults and less than 3% in children [6]. In Malaysia, the reported incidence of brain and nervous system tumours is 2.9% among males and 1.9% in females, however further subdivisions of specific types of tumours are not delved into [2]. Being a rare tumour presentation, early recognition and intervention benefit the patient and provide a better prognosis [7]. 

Pineal gland tumours can be classified according to the appearance of solidity with the presence of calcification or cystic lesions. It can also be further subdivided according to its histology, based on the World Health Organization Classification. The pineal gland tumour can be divided into four major groups with germinal tumour accounting for 50% of pineal gland tumours. Listed according to increasing malignancy potential are mature or benign teratoma, germinoma, immature or malignant teratoma, embryonic carcinoma, yolk sac tumour and choriocarcinoma. Pineal parenchymal tumours comprising 30% of all pineal gland tumours can be histologically seen as pineocytoma, pineoblastoma and pineal parenchymal tumours of intermediate differentiation. Glial and other tumours from supporting stroma comprise 25% of the tumours, papillary tumour of the pineal gland being its subtype and pineal cyst accounting for less than 3%, usually presenting as asymptomatic lesions and coincidental findings [8-10]. In Asia, the most prevalent type of brain tumour is germinoma, contributing to nearly 50% of all brain tumours [11].

Germ cell tumour has a male-to-female predominance with a ratio of 11:8, and the common age of occurrence is around 16 years old [12]. Common presentations are compressive symptoms; hydrocephalus, headache, vomiting, aqueduct stenosis, and compressive hypothalamic symptoms such as diabetes insipidus and stunted growth [13]. Other symptoms depend on the region of compression; if superior cerebellar peduncles are affected the patient may present with ataxia and dysmetria [4]. Locations of germ cell tumours can differ intracranially. The most common locations are the supra-sellar, pineal and basal ganglia regions [5]. In a retrospective study of 153 histologically confirmed germ cell tumours, nearly 50% of the tumour were located in the pineal region, followed by 30% in neurohypophysis and less than 3% in the basal ganglia. In accordance with this, the pineal gland was noted to be the most common tumour location in another study of 181 cohorts, where nearly 45% of tumours were located at the pineal gland, 21% at the suprasellar region and 16% at the basal ganglia. There were also bifocal presentations at both suprasellar and pineal regions in 13% of the cases [7]. As shown in our case reported above, the lesion was of pineal gland location with drop metastases in the fourth ventricle, constituting one of the rare locations of metastases.

Ophthalmological symptoms that are commonly associated with pineal gland tumours are known as Parinaud syndrome. This was first described in 1800 by French ophthalmologist, Henri Parinaud. It was initially speculated that the eye findings are due to lesions in the quadrigeminal area. Other terms used for this condition were Sylvian aqueduct syndrome, dorsal midbrain syndrome, pretectal syndrome, and Koerber-Salus-Elschnig syndrome.

Parinaud syndrome is characterised by a triad of paralysis of conjugate up gaze, light-near dissociation of the pupils and convergence-retraction nystagmus during upward saccades. Lid retraction in the primary gaze (Collier’s sign) can also be observed [14]. Limitations of the conjugate up gaze are due to the involvement of the vertical gaze centres, being in close proximity to the superior colliculus. The fibres of the upward gaze are directed laterally from the medial longitudinal fasciculus and decussate in the posterior commissure, this being anatomically more at risk from the pressure effect of space-occupying lesions. The other highly localizing sign of the Parinaud syndrome is the convergence retraction nystagmus which is more prominent in the upward gaze. This is caused by the damage to the supranuclear fibres which inhibits the mid-brain convergence and divergence neurons. In presence of this sign, lesions of the dorsal midbrain must be suspected. Parinaud syndrome is seen in 65% of other primary midbrain lesions such as strokes, haemorrhages and neoplasm and is reported to be seen in 30% of pineal gland tumours with pressure effect on the dorsal midbrain [14]. In our case, the patient presented with the classical triad of Parinaud syndrome, as reported where 65% of patients present with this classical presentation [15].

Diagnosis of pineal gland tumours depends on three major parameters: clinical presentation, imaging modalities, and histopathology with serum marker and cerebral-spinal fluid marker as adjuncts to the main parameters [13]. In terms of imaging, the selection of MRI would be the gold standard as exposing the patient to repeated computerised tomography increases the exposure to radiation and increases the risk of malignancy. MRI scans are able to give detailed images and are able to differentiate between the pineal and para-pineal invasion. For germinoma of the pineal gland, the findings are not specific and can present as a well-delineated, ovoid, lobulated homogenous mass with engulfing of the normal physiological calcification. The pattern of the calcification can also give more indication as to which tumour type is being dealt with: engulfed calcification is present in germinoma, distributed calcification is present in pineal parenchymal tumour and in pineoblastoma, there is an infiltration of the surrounding structures by the calcification [1]. The ability to differentiate between germinoma and other pineal gland tumours based on its degree of heterogeneity, enhancement and T1W1 and T2W1 intensity values are of clinical dilemma and does not differentiate between both entities [16]. The presence of drop metastases is the seeding into the subarachnoid space with resultant intradural extramedullary metastases [1]. This has been reported in nearly 30% of the pineal gland tumour cases, with medulloblastoma being the most commonly affected subtype constituting nearly 50% of all cases, followed by ependymomas and germinomas and less likely by teratomas. This is speculated due to the absence of the blood-brain barrier in the pineal glands [13,17]. As shown in our case, there were drop metastases in the fourth ventricle. Drop metastases are not looked for as a prognostic value but findings in the later stage after chemotherapy and radiotherapy indicate resistance to the adjuvant therapy and have poorer prognosis [1,16].

As mentioned earlier, biochemical serum and cerebrospinal fluid markers act as adjuncts to the diagnostic parameters. In pineal gland tumours, for embryonal and immature teratomas, there can be an increase of AFP in both serum and in the cerebro-spinal fluid. In choriocarcinoma and germinoma, there are increased levels of the b-HCG, lactate dehydrogenase and placenta alkaline phosphatase [8,13]. In our case report above, all the serum markers were in the normal range except CEA, which was slightly elevated.

## Conclusions

Patients with pineal gland tumours can present with various presentations and early diagnosis and management can help with the prognosis of the tumour. As clinical presentations can be vague, other adjuncts such as tumour markers, liver markers, cerebral spinal fluid markers, MRI imaging to look for tissue involvement and extension and tissue biopsy are needed for a definite diagnosis. As pineal gland tumours comprise a small number of cases in our current population, this has to be given some importance as it affects the younger age group and their quality of life in the long run. As shown in our case, the development of the tumour at the time of presentation was of a short duration of four months with acute deterioration, the initial presentation was vague and the patient presented with motor symptoms rather than typical pineal gland tumour symptoms. A high index of suspicion is needed to diagnose these cases early as prompt intervention promises a better outcome.
